# miRNA-186-5p inhibits migration, invasion and proliferation of breast cancer cells by targeting SBEM

**DOI:** 10.18632/aging.204887

**Published:** 2023-07-20

**Authors:** Hui Hao, Bingsheng Wang, Lin Yang, Yinzhou Sang, Wei Xu, Wei Liu, Lili Zhang, Da Jiang

**Affiliations:** 1 Department of Medical Oncology, The Forth Hospital of Hebei Medical University, Shijiazhuang 050000, China; 2 Department of Medical Oncology, Hebei Cangzhou People’s Hospital, Cangzhou 061001, China; 3 Graduate School, Chengde Medical University and Cangzhou People’s Hospital, Cangzhou 061001, China; 4 Department of Pathology, Hebei Cangzhou People’s Hospital, Cangzhou 061001, China; 5 Department of Medicine, Cangzhou Medical College, Cangzhou 061011, China

**Keywords:** breast cancer, miR186-5p, migration, invasion, proliferation

## Abstract

The paper aimed to investigate the effect of miR186-5p on invasion and migration of breast cancer cells and its molecular mechanism. MicroRNA-186-5p was found to be low expressed in breast cancer and highly expressed in SBEM by bioinformatics analysis. After transfecting MDA-MB-231 cells with miR-186-5p inhibitor NC, miR-186-5p inhibitor, miR-186-5p mimic NC and miR-186-5p mimic, respectively. The migration and invasive ability of breast cancer cells were detected by cell scratch test and Transwell test. Moreover, after adding 740 Y-P to the miR-186-5p mimic NC group and miR-186-5p mimic group cells, SBEM and PI3K pathway-related proteins were detected by Western blotting and proliferation of the cancer cells was evaluated by monoclonal cell experiment. Meanwhile, exogenous miR-186-5p mimic in MDA-MB-231 cells significantly inhibited the expression of SBEM, p-PI3K, p-AKT and their downstream pathways, MMP1, MMP3, MMP9, CyclinD1, PCNA and CyclinB1 proteins and reduced proliferation of breast cancer cells. Furthermore, the expression of SBEM protein in the miR-186-5p mimic + 740Y-P group was significantly lower than the miR-186-5p mimic NC + 740Y-P group after adding 740 Y-P. However, there were no significant changes in the protein’s levels associated with PI3K pathway and the cancer cells proliferation. These results suggest that low expression of miR-186-5p in breast cancer results in an abnormally high expression of SBEM, activation of PI3K/AKT signaling pathway, promoting migration and invasion of human breast cancer cells.

## INTRODUCTION

Breast cancer is one of the most common malignant tumors in women, which seriously threatens women’s health [1]. Invasion and metastasis are important biological features of malignant tumors, closely related to breast tumor recurrence and poor prognosis, and a major cause of patient death [2]. However, the molecular mechanism driving metastasis of breast cancer is still unknown [3]. Therefore, further research on breast cancer metastasis mechanism that provides the theoretical basis for developing new breast cancer diagnosis and treatment schemes is critical for improving the survival rate and outcome of patients with breast cancer. Micro RNA (miRNA), as an oncogene and tumor suppressor, plays an important role in many cancers [4, 5].

miRNA, which is highly conserved among species, is a non-protein coding single-stranded RNA molecule with 18 to 25 nucleotides encoded by endogenous genes [6]. Dysregulation of miRNAs results in abnormal expression of many genes, which is associated with the onset and progress of various human diseases, including cancers [7]. The mechanism of miRNA in the regulation of breast cancer metastasis includes regulating epithelial mesenchymal transition [8], characteristics of cancer stem cells [9], tumor microenvironment [10], angiogenesis [11], as well as exosomes [12]. For example, miR-140-5p inhibits angiogenesis and invasion of breast cancer cells by regulating VEGF-A [13] and miR-204-5p lowers the proliferation and migration of breast cancer cells through remodeling the tumor immune microenvironment [14], indicating that the abnormal mi RNA in breast cancer cells not only affects the occurrence and progress of breast cancer, and maybe an effective biomarker and target for breast cancer metastasis evaluation and biotherapy [15]. In this study, the low expression of miRNA-186-5p in breast cancer was found by TCGA database analysis, but the biological role of miRNA in breast cancer has not yet been reported. As an important component of epigenetics, miRNAs bind to complementary binding sites on the 3′-untranslated regions (3′-UTRs) of the target gene mRNA, resulting in degradation or translation inhibition of mRNA [4]. Therefore, we predicate the potential target genes of miRNA-186-5p by bioinformatics and found that small breast epithelial mucin (SBEM) is the most likely potential target gene. As a secretory protein of MUC family [16], SBEM is mainly expressed in breast and salivary glands, and is highly expressed in breast cancer tissues and metastatic lymph nodes [17]. Moreover, SBEM is a recognized breast-specific gene and is considered to be a promising breast-specific marker [18]. Based on these research backgrounds, it is presumed that miRNA-186-5p may play a significant role in the invasion and metastasis of breast cancer cells by targeting SBEM.

In this study, bioinformatics technology was applied for enrichment and pathway analysis. Furthermore, the specific regulation mechanism of miRNA-186-5p/SBEM on invasion and metastasis of breast cancer cells was explored by basic cell experiments, which may provide potential biomarkers for clinical evaluation of invasion and metastasis of breast cancer cells.

## MATERIALS AND METHODS

### Bioinformatics analysis

GSE139038, data set of breast cancer-related gene expression was searched and downloaded from the GEO (GENE EXPRESSIONOMNIBUS) database (https://www.ncbi.nlm.nih.gov/gds) and data set GSE143564 containing the miRNA sequencing data set of patients with breast cancer was downloaded. Quantile-normalization and differential gene analysis of RNA-seq data were performed using R language limma software package (|logFC| < 1, *p*-value < 0.05). The volcano plot of visible differentially expressed genes (DEGs) from the GSE139038 data set was created in R using the ggplot2 package. The intricate heat map of DEGs was created using the R package pheatmap. Similarly, a volcano plot of visualized DEGs was generated, as well as complicated heat map of the data set GSE143564.

### Functional enrichment analysis

The DEGs in the data set GSE139038 were subjected to GO (Gene Ontology) enrichment and KEGG (Kyoto Encyclopedia of Genes and Genes) enrichment analysis. The DEGS corresponding to biological processes, cellular components, and molecular functions were analyzed with DAVID online database tool (https://david.ncifcrf.gov) to integrate GO terminology and create a network of biological processes of DEGs. The GO pathway map and KEGG pathway enrichment map of DEGs were plotted in R language environment using Goplot and gggplot2 package.

### Gene set enrichment analysis (GSEA)

GSEA tool (https://www.gsea-msigdb.org) was used to carry out GSEA and plot the GSEA pathway map.

### Prediction of miRNA target gene

miRNA candidate target genes were predicted by miRDB and targetScan online tool, and target gene was predicated with Venn diagram mapped together with GSE143564 differential genes. Besides, the binding sites of mRNA and miRNA were mapped according to gene prediction results.

### Cell culture and transfection

The human breast cancer cell lines: MDA-MB-231, SUM159PT, JIMT-1, ZR-75-30 were purchased from the American Type Culture Collection and used within 6 months after purchase. MDA-MB-231 cells were cultured in DMEM supplemented with 10% fetal bovine serum. All cells were cultured in a sterile constant-temperature incubator containing 5% CO_2_ at 37°C, and the medium was replaced every 2–3 d. These cells were passaged when they reached a confluence of 80–90%. The experiments were conducted when the cells were in a logarithmic growth phase. MDA-MB-231 cells were transfected with 50 nM miR-186-5p inhibitor NC, miR-186-5p inhibitor NC, miR-186-5p mimic NC and miR-186-5p mimic (Suzhou GenePharma, China), respectively, following the manufacturer instructions. Cells were collected 48 h after transfection and 740 Y-P (agonist of PI3K) extracted proteins were added to miR-186-5p mimic NC and miR-186-5p mimic cells for subsequent experiments. miR-186-5p mimic sequences: (5′-caa aga auu cuc cuu uug ggc u-3′); miR-186-5p mimic NC: (5′-cag uac uuu ugu gua gua caa a-3′); miR-186-5p inhibitors: (5′-agc cca aaa ggagaa uuc uuu g-3′); miR-186-5p inhibitor NC: (5′-uuu gua cua cac aaa agu acu g-3′).

### Wound healing assay

The migration ability of human breast cancer cell lines: MDA-MB-231, SUM159PT, JIMT-1, ZR-75-30 cells was determined by wound healing assay. Cells were collected 48 h after transfection, and a total of 1 × 10^6^ MDA-MB-231 cells were seed to each well of a six-well plate and cultured till 90% confluence. Tip of 10 μl pipette was used to scrape the plate to create a wound in a central area of the cell monolayer. The medium was discarded and the plate was washed twice with PBS. Then, DMEM without serum was added to each well, and images of the migration area were observed an optical microscope at 0 h, 24 h and 48 h, respectively. The scratch distance was measured with Image J software.

### Transwell migration and invasion test

Cells were collected 48 h after transfection for Transwell assay. A total of 40 μL of diluted Matrigel gum (volume ratio of serum-free medium to Matrigel gum is 1: 8) was previously added to the filter membrane of the Transwell chamber in the invasion test, which was neglected in the migration test. The cells of each group were collected by digestion and centrifugation, and then counted after being resuspended in serum-free media and thoroughly mixed, and the concentration was adjusted to 2 × 10^5^ /mL. With 3 multiple wells for each group, the lower chamber of Transwell (Bottom of the 24-well plate) was added with 500 μL of medium containing 10% FBS, and the upper chamber was supplemented with 200 μL of serum-free cell suspension containing 3.0 × 10^4^ cells and both chambers were incubated in the incubator containing 5% CO_2_ for at 37°C for 13 h. Then, the chambers were taken out, washed with PBS buffer and added with 4% paraformaldehyde for fixing cells for 15 min, wiped with a wet cotton swab to remove the cells, dried, and stained with 0.5% crystal violet for 5 min, washed with PBS and dried. Under a positive phase contrast optical microscope, five visual fields (100×), left, right, upper, lower and middle, were photographed, and the average value of the five visual fields was calculated to determine the invasion and migration ability of cells in each well.

### Western blot

Cells were collected 48 h after transfection and inoculated into 96-well culture plates. 740 y-p (25 nM) was added to NC group and miR-186-5p mimic group. It was divided into four groups: miR-186-5p mimic NC group, mir-186-5p mimic group, miR-186-5p NC+740 Y-P group and miR-186-5p mimic+740 Y-P group, as well as, each group had 5 multiple Wells. After 48 hours of incubation, RIPA lysate and phenylmethanesulfonyl fluoride (PMSF) were used to extract protein samples from the cells. The protease inhibitor PMSF was added according to PMSF: RIPA = 1:100 to lyse cells on ice for 30 min. Cells were centrifuged at 12000 rpm at 4°C for 15 min and the supernatant was transferred to a 200 μl EP tube and stored at 20°C. The concentration of protein in the supernatant was detected by BCA kit. The loading amount of the sample was calculated according to the protein concentration of the sample and the loading system, ultrapure water and 5× loading buffer were added to the sample and denatured in a metal bath at 100°C for 10 min after mixing and centrifugation. The proteins were separated by SDS-PAGE (sodium dodecyl sulfate-polyacrylamide gel electrophoresis), and the target proteins with different molecular weights were transferred to the PVDF membrane and blocked in 5% defatted milk at room temperature for 2 h. The PVDF membrane was incubated in primary anti-SBEM, p-PI3K, p-AKT, vimentin, N-cadherin, MMP1, MMP3, MMP9, CyclinD1, PCNA, CyclinB1 and GAPDH (dilution ratio 1:1000), respectively, overnight at 4°C. Then, the PVDF membrane was washed 3 times with PBST on a shaker for 10 min/time and incubated with horseradish peroxidase-labeled goat anti-rabbit or goat anti-mouse IgG secondary antibody (dilution ratio 1: 5000) for 2 h at room temperature. Finally, the high-sensitivity ECL chemiluminescence kit and FluorChem Q instrument were used for exposure and the AlphaView software system was applied for protein quantitative analysis.

### Monoclonal proliferation test

After trypsin digestion of the cells in logarithmic growth phase, the complete medium (basal medium + 10% fetal bovine serum) was resuspended to be suspension and counted. In a six-well plate, 400–1000 cells/well (700 cells/well, depending on cell growth) of each test group were seeded, and incubated for 14 days or until the number of cells in most single clones was greater than 50. In the process, the medium was changed every three days and cell state was observed. After cloning, the cells were photographed under a microscope, and then washed with PBS once, 1 mL of 4% paraformaldehyde was added to each well to fix cells 30–60 min, and then washed with PBS once. 1 ml of crystal violet solution was added to each well to stained cells for 10–20 min. The cells were then washed multiple times with PBS, dried, and positive clones were seen under a microscope, with each clone containing more than 50 cells, and then photographed with a camera. The number of clones (about 0.3–1.0 mm) was counted to determine the clone formation rate and the clone size.

### Nude mouse tumorigenesis

MDA-MB-231 cells in logarithmic growth stage were digested with trypsin, centrifuged and adjusted to a density of 2.5 with serum-free medium × 10^7^/ml, stored at 4°C for standby. Nude mice, aged 4–6 weeks and weighing 18–20 g, were purchased from Beijing Vital River Laboratory Animal Technology Co. Ltd., were randomly divided into 2 groups: miR-186-5p NC group and miR-185-5p mimic group, *n* = 6 for each group. Each nude mouse was subcutaneously injected with 200 μl in the middle and lower part of the armpit. 2 weeks later, when the tumor volume reached 50 mm^3^, mir-186-5p NC and mir-186-5p mic were injected into the caudal vein, 20 nmol each time, with an injection volume of 0.1 ml, once every 3 days, and repeated for 4 weeks. All experiments were approved by the Ethics Committee of Cangzhou People’s Hospital.

### Statistical analysis

Rv3.6.1 software package DEseq2 and ggpubr software package were used for statistical analysis of bioinformatics. Differential gene analysis was performed with the Wald test. The cytokines between the two groups were compared using rank-sum test. The data were analyzed with GraphPad Prism 7.0 and expressed as mean ± standard deviation. The difference between the two groups was analyzed using Student’s t. The differences between groups were analyzed by one-way ANOVA. *P* < 0.05 was considered statistically significant. All the tests in this study were independently triplicated.

## RESULTS

### Screening of DEGs

**Figure 1:**
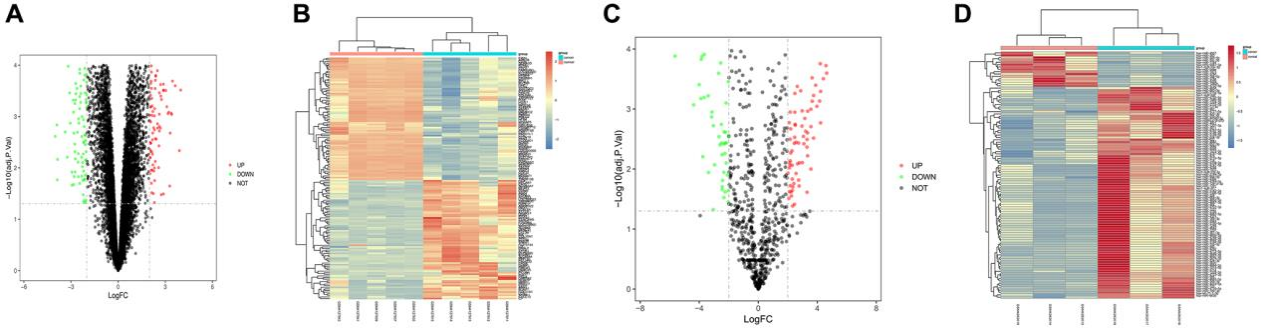
**Screening of differentially expressed genes (DEGs).** (**A** and **B**) DEG volcanic map and cluster analysis heat map of GSE139038; (**C** and **D**) DEG volcanic map and cluster analysis heat map of GSE143564.

The data set GSE139038 related to breast cancer was downloaded from GEO database, and the data were processed by quantile normalization, and GSE139038 was screened according to the standards of *p*-value < 0.05 and |logFC| < 2. Our results suggested that there were 154 DEGs in the mRNA of breast cancer, of which 76 were up-regulated and 78 down-regulated. The volcano plot (Figure 1A) of DEGs of the data set GSE139038 was constructed in R software using the software package ggplot2, and the complex heat map of DEGs (Figure 1B) was constructed using R software package pheatmap. Similarly, the volcano plot of visualized DEGs (Figure 1C) and the complex heat map of the data set GSE143564 (Figure 1D) were constructed.

**Table:** Detailed information for heat maps in Figure 1.

	**logFC**	**AveExpr**	**t**	***P*-val**	**adj.*P*.Val**	**B**
GJB2	5.91	2.60	9.73	0.00	0.02	3.81
CLEC3A	5.84	2.31	6.67	0.00	0.21	1.77
CGA	5.11	−3.99	4.20	0.00	0.39	−0.94
MMP11	4.23	2.30	4.95	0.00	0.33	0.00
MMP13	4.16	0.87	5.16	0.00	0.33	0.24
MUCL1	3.95	−4.47	4.15	0.00	0.39	−1.01
MATN3	3.87	−1.23	4.70	0.00	0.33	−0.30
LRRC15	3.47	0.47	4.17	0.00	0.39	−0.99
LUM	2.82	4.16	4.99	0.00	0.33	0.04
HIST1H2AM	2.53	−1.97	4.00	0.00	0.39	−1.20
FN1	2.48	−1.45	4.27	0.00	0.39	−0.85
HIST1H1C	2.41	−0.68	4.21	0.00	0.39	−0.93
CST1	2.26	−0.13	4.06	0.00	0.39	−1.13
TEX13A	2.25	−1.60	4.36	0.00	0.37	−0.73
ST6GAL2	2.22	2.54	4.62	0.00	0.33	−0.41
MLF1IP	2.02	−2.02	4.60	0.00	0.33	−0.43
AEBP1	2.02	2.38	4.37	0.00	0.37	−0.72
SHCBP1	2.02	−2.86	4.11	0.00	0.39	−1.07
SDCCAG1	−2.04	−1.11	−4.10	0.00	0.39	−1.07
CYR61	−2.09	0.87	−4.08	0.00	0.39	−1.10
KCNK2	−2.24	−0.53	−5.25	0.00	0.32	0.35
CIDEC	−2.28	1.12	−4.18	0.00	0.39	−0.97
TXNIP	−2.32	2.30	−5.26	0.00	0.32	0.36
EDNRB	−2.34	−0.77	−4.15	0.00	0.39	−1.01
CRYAB	−2.40	3.25	−5.86	0.00	0.32	1.00
KBTBD11	−2.50	−0.55	−4.44	0.00	0.36	−0.63
MT1E	−2.54	0.70	−4.57	0.00	0.34	−0.46
MAMDC2	−2.61	1.97	−4.25	0.00	0.39	−0.87
AKR1C1	−2.63	−1.80	−4.24	0.00	0.39	−0.89
MRAP	−2.66	0.88	−4.12	0.00	0.39	−1.05
ZBTB16	−2.84	2.38	−4.76	0.00	0.33	−0.23
KIAA1045	−2.92	0.40	−5.61	0.00	0.32	0.74
RASD1	−2.96	1.58	−5.34	0.00	0.32	0.45
STYK1	−2.97	−0.10	−4.02	0.00	0.39	−1.19
KLF4	−2.98	0.92	−5.04	0.00	0.33	0.11
FLJ30046	−3.01	−0.62	−4.42	0.00	0.36	−0.65
FMO2	−3.12	2.08	−7.20	0.00	0.16	2.20
GSTA1	−3.30	0.62	−4.00	0.00	0.39	−1.21
NR3C2	−3.42	2.52	−4.21	0.00	0.39	−0.93
PKHD1L1	−3.46	0.30	−5.08	0.00	0.33	0.15
DUSP1	−3.53	1.62	−4.53	0.00	0.35	−0.52
DENND2A	−3.60	−0.46	−4.77	0.00	0.33	−0.22
AKR1C2	−3.60	−1.24	−4.16	0.00	0.39	−0.99
TMEM100	−3.85	−0.25	−5.66	0.00	0.32	0.79
SDPR	−3.95	1.42	−5.52	0.00	0.32	0.64
SEL1L2	−4.09	1.46	−5.35	0.00	0.32	0.45
ADH1A	−4.18	3.56	−5.46	0.00	0.32	0.57
EGR1	−4.29	0.75	−5.67	0.00	0.32	0.80
PLIN	−4.60	2.35	−5.36	0.00	0.32	0.47
FOS	−5.06	1.91	−4.77	0.00	0.33	−0.22
	**logFC**	**AveExpr**	**t**	***P* val**	**adj.*P*.Val**	**B**
hsa-miR-184	5.44	2.57	21.48	0.00	0.00	3.65
hsa-miR-503-5p	4.44	2.94	8.66	0.00	0.12	1.42
hsa-miR-375	3.04	6.27	8.52	0.00	0.12	1.36
hsa-miR-200a-5p	2.71	1.87	8.39	0.00	0.12	1.30
hsa-miR-7975	2.47	3.74	8.04	0.00	0.13	1.14
hsa-miR-21-5p	5.86	3.87	7.62	0.00	0.14	0.92
hsa-miR-3663-3p	2.29	2.23	7.09	0.00	0.19	0.63
hsa-miR-425-5p	3.45	6.85	6.91	0.00	0.19	0.52
hsa-miR-5100	2.25	6.67	6.71	0.00	0.20	0.39
hsa-miR-1254	1.32	0.84	6.17	0.00	0.29	0.03
hsa-miR-185-5p	1.75	8.13	5.89	0.00	0.33	−0.17
hsa-miR-4632-5p	-1.38	4.79	−5.80	0.00	0.33	−0.24
hsa-miR-378f	-1.99	5.43	−5.72	0.00	0.33	−0.30
hsa-miR-141-3p	2.73	2.00	5.66	0.00	0.33	−0.35
hsa-miR-130b-3p	1.53	4.62	5.47	0.00	0.35	−0.50
hsa-miR-342-3p	2.01	9.93	5.46	0.00	0.35	−0.51
hsa-miR-186-5p	3.09	7.44	5.32	0.00	0.38	−0.62
hsa-miR-25-5p	2.29	2.13	5.16	0.00	0.39	−0.76
hsa-miR-196a-5p	5.32	3.68	5.12	0.00	0.39	−0.79
hsa-miR-6086	−1.50	2.87	−5.10	0.00	0.39	−0.81
hsa-miR-378c	−1.93	6.65	−5.05	0.00	0.39	−0.85
hsa-miR-3615	1.80	0.82	5.05	0.00	0.39	−0.86
hsa-miR-378i	−2.42	5.54	−4.97	0.00	0.40	−0.92
hsa-miR-3176	1.29	0.73	4.90	0.00	0.40	−0.99
hsa-miR-483-5p	−3.59	4.41	−4.87	0.00	0.40	−1.01
hsa-miR-381-3p	1.09	0.98	4.77	0.00	0.40	−1.11
hsa-miR-342-5p	1.86	6.68	4.71	0.00	0.40	−1.16
hsa-miR-3910	−1.06	0.48	−4.68	0.00	0.40	−1.20
hsa-miR-181b-5p	2.33	6.43	4.66	0.00	0.40	−1.21
hsa-miR-484	3.10	1.90	4.64	0.00	0.40	−1.23
hsa-miR-4800-3p	1.23	0.59	4.64	0.00	0.40	−1.23
hsa-miR-330-3p	2.23	2.22	4.60	0.00	0.41	−1.28
hsa-miR-4649-5p	−1.05	5.31	−4.57	0.00	0.41	−1.30
hsa-miR-409-3p	2.63	3.49	4.54	0.00	0.41	−1.33
hsa-miR-663b	1.05	0.77	4.48	0.00	0.42	−1.39
hsa-miR-769-3p	1.49	0.51	4.35	0.00	0.45	−1.52
hsa-miR-7977	2.87	5.40	4.23	0.00	0.47	−1.64
hsa-miR-4513	1.13	0.90	4.23	0.00	0.47	−1.64
hsa-miR-500a-5p	1.92	2.13	4.15	0.01	0.47	−1.72
hsa-miR-100-5p	−1.33	8.92	−4.07	0.01	0.47	−1.81
hsa-miR-21-3p	1.92	1.61	4.05	0.01	0.47	−1.83
hsa-miR-183-3p	2.24	1.52	4.04	0.01	0.47	−1.84
hsa-miR-512-3p	1.05	0.21	4.03	0.01	0.47	−1.85
hsa-miR-320e	−1.36	7.74	−3.99	0.01	0.47	−1.90
hsa-miR-24-2-5p	2.28	1.46	3.97	0.01	0.47	−1.92
hsa-miR-4738-3p	1.29	0.74	3.97	0.01	0.47	−1.92
hsa-miR-4286	2.37	1.96	3.95	0.01	0.47	−1.94
hsa-miR-422a	−1.50	5.09	−3.94	0.01	0.47	−1.95
hsa-miR-4417	3.14	2.98	3.92	0.01	0.47	−1.97
hsa-miR-421	2.23	1.49	3.92	0.01	0.47	−1.97

### Bioinformatics analysis

**Figure 2:**
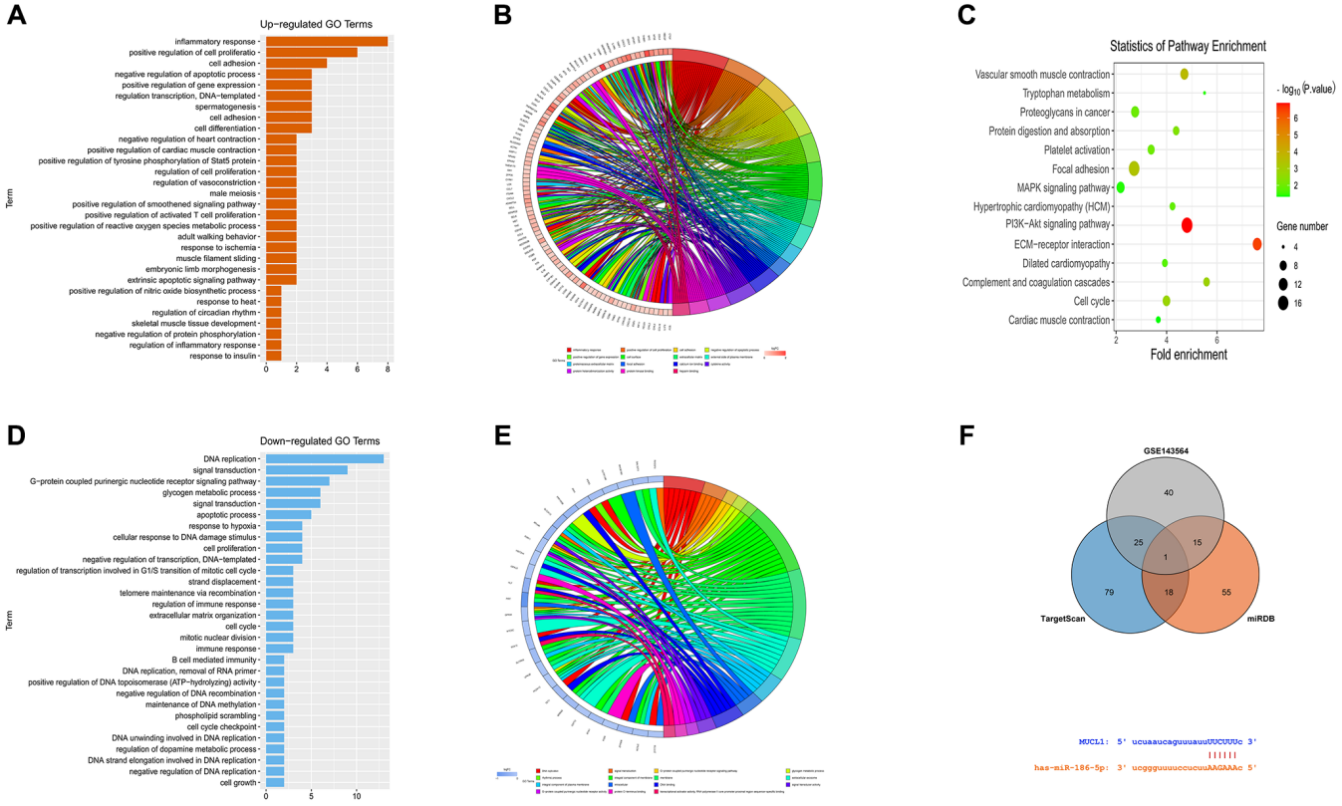
**Enrichment analysis of GSE139038.** (**A** and **B**) GO up-regulated pathway diagram. (**C**) KEGG pathway. (**D** and **E**) GO down-regulated pathway diagram. (**F**) Venn diagram, mRNA and miRNA binding site diagram.

**Figure 3:**
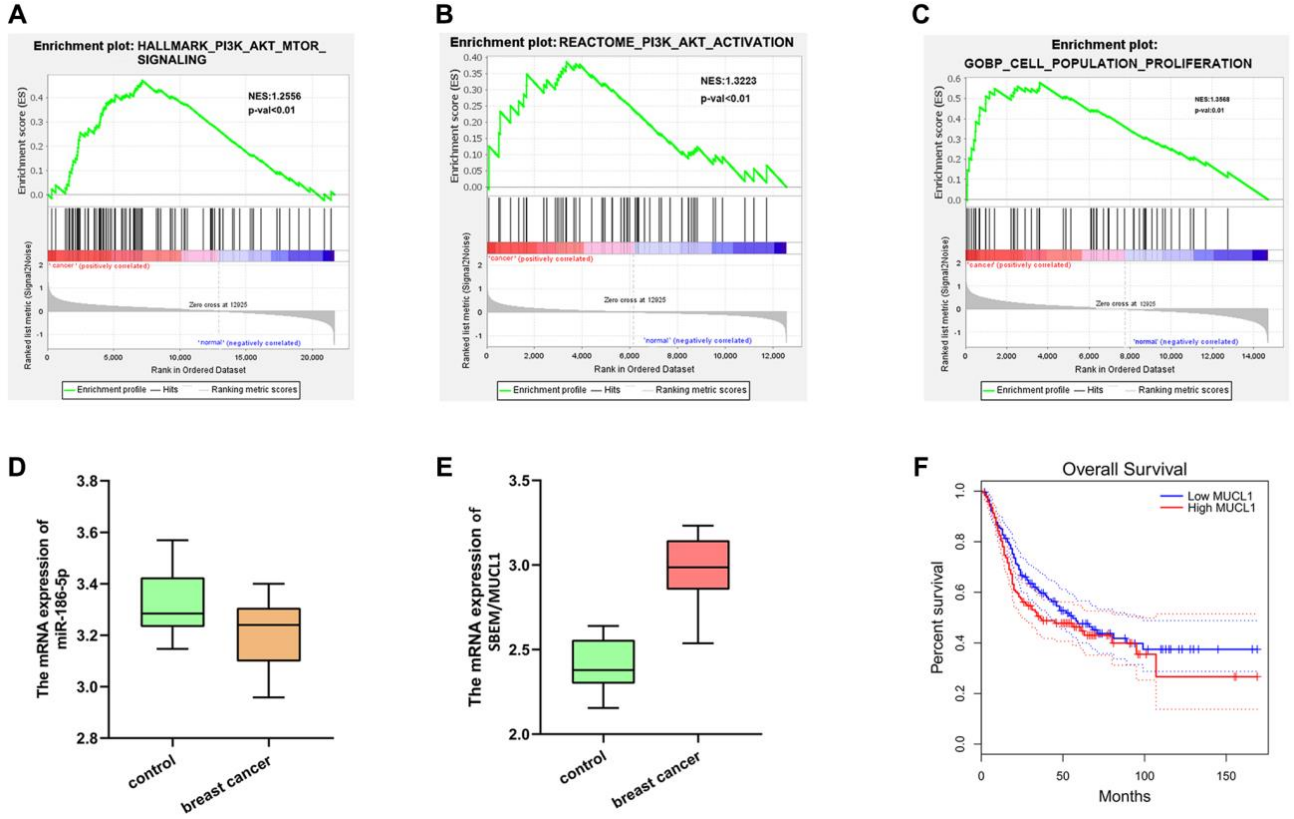
**GSEA analysis and target gene statistical analysis.** (**A**–**C**) GSEA analysis showed that the PI3K-Akt-MTOR signaling pathway and cell proliferation pathway were enriched; (**D**) Low expression of Mir-186-5p in breast cancer; (**E**) High expression of SBEM (MUCL1) in breast cancer; (**F**) Survival curve plotted with low survival associated with high expression of SBEM.

The DEGs in the data set GSE139038 were subjected to GO enrichment and KEGG enrichment analysis. The DEGs corresponding to biological processes were analyzed using DAVID online database tool (https://david.ncifcrf.gov) to integrate GO terminology and create a network of biological processes of DEGs. The GO mainly has three functions, including biological process (BP), cellular component (CC) and molecular function (MF). The up-regulation pathway map (Figure 2A and 2B) and down-regulation pathway map (Figure 2D and 2E) of the DEGs were mapped using R language. Using the GO path diagram, we found that inflammatory response, positive regulation of cell proliferation and cell adhesion these up-regulated pathways as well as DNA replication, signal transduction and G-protein-coupled purinergic receptor signaling these down-regulated pathways were enrichment pathways of breast cancer. Differential genes were applied to analyze the KEGG pathway and plot the KEGG pathway map (Figure 2C). We found that the signaling pathways, such as the PI3K-Akt signaling pathway and cell metastasis were enriched. GSEA analysis showed that the PI3K-AKT-MTOR signaling pathway and cell proliferation pathway were enriched (Figure 3A–3C).

### Prediction of miRNA target gene

miRNA candidate target genes were predicted by miRDB and TargetScan online tool, and together with GSE143564 differential genes. Venn diagram was plotted with VennDiagram package to take the intersection, to find the co-bound miR-186. Besides, mRNA and miRNA binding sites were plotted according to gene predication results (Figure 2F).

### Statistical analysis of target genes

Based on the GEO and TCGA databases, breast cancer-related genes analysis\showed that miR-186-5p was low-expressed (Figure 3D), and SBEM (MUCL1) was high-expressed in the breast cancer group (Figure 3E) simultaneously, the low survival rate associated with high-expressed SBEM was plotted (Figure 3F).

### Effect of miR-186-5p on the migration of breast cancer cells

**Figure 4:**
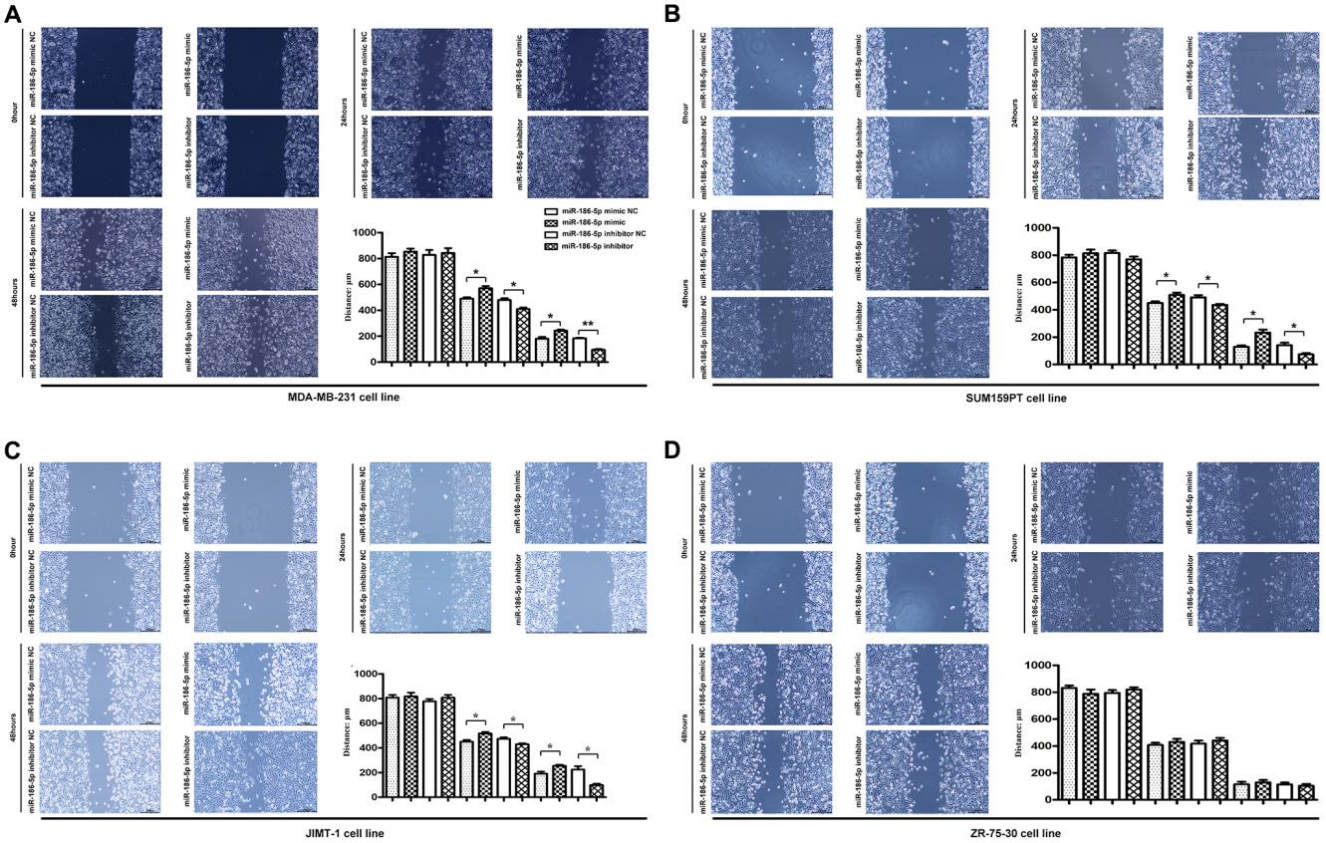
**Scratch assay was used to detect the migration ability of MDA-MB-231, SUM159PT, JIMT-1, but not ZR-75-30 cells in each group.** (**A**–**D**) showed the image of the migration area 0 h, 24 h and 48 h after the scratch manufacturing, respectively. Statistical results of scratch distance of MDA-MB-231 cells in each group. ^*^P < 0.05 and ^**^P < 0.01 indicated statistically significant differences.

**Figure 5:**
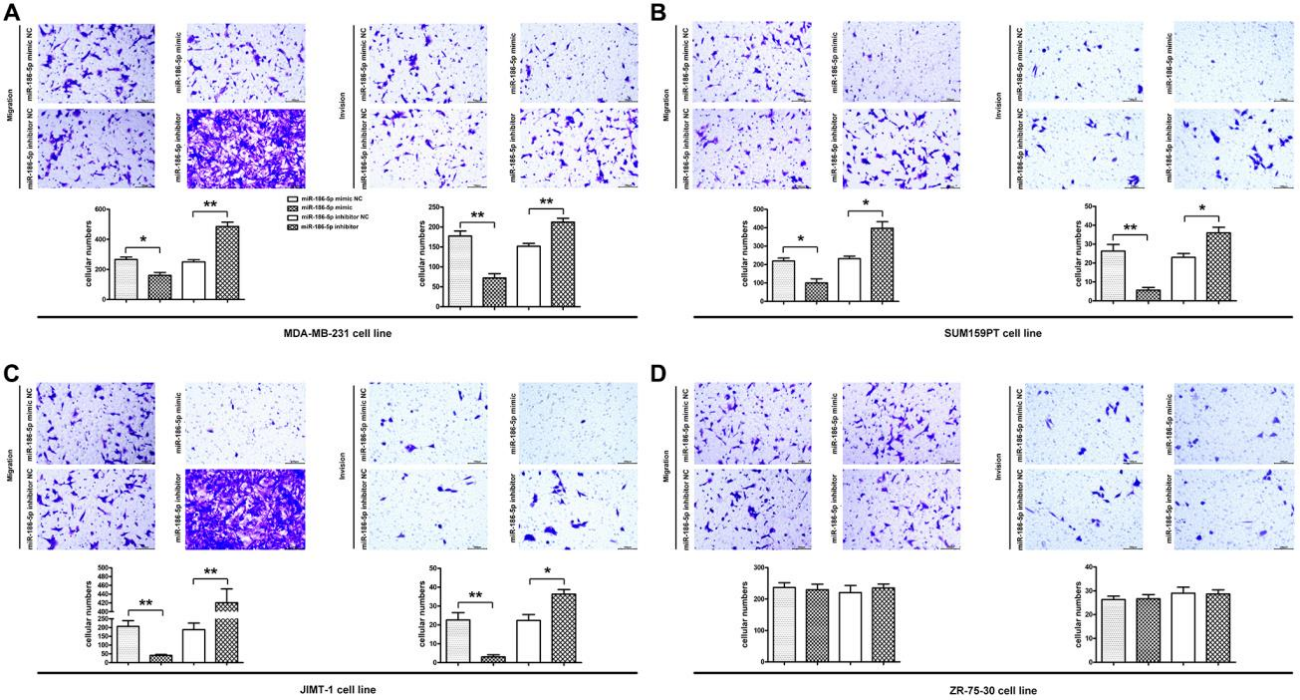
**Migration and invasion ability of MDA-MB-231, SUM159PT, JIMT-1, but not ZR-75-30 cells in each group.** (**A**–**D**) Migration was used to detect the number of MDA-MB-231 cells passing through the lower compartment (×400); Invasion was performed to detected the number of mDA-MB-231 cells that crossed the basement membrane (×400). ^*^P < 0.05 and ^**^P < 0.01 indicated statistically significant differences.

Scratch test and Migration test were performed to explore the migration ability of MDA-MB-231, SUM159PT, JIMT-1 cells. The results of the scratch test showed that the cell scratch distance of the miR-186-5p inhibitor group was significantly lower than the miR-186-5p inhibitor NC group at 24 h and 48 h after cell transfection (Figure 4A–4D), indicating that the migration distance of tumor cells to the center of scratch was increased. On the contrary, the scratch distance of MDA-MB-231 cells in miR-186-5p mimic group was significantly longer than miR-186-5p mimic NC group, indicating that miR-186-5p mimic inhibited the migration of tumor cells. Similarly, the migration results showed that the number of migrated MDA-MB-231, SUM159PT, JIMT-1 cells was increased and decreased significantly after transfecting cells with miR-186-5p inhibitor and miR-186-5p mimic, respectively, compared to the miR-186-5p inhibitor NC group and the miR-186-5p mimic NC group (Figure 5A–5D).

### Effect of miR-186-5p on the invasion of breast cancer cells

The invasion of MDA-MB-231 cells was further verified by Invasion test. Figure 5B showed that the number of MDA-MB-231 cells crossing the basement membrane in the miR-186-5p inhibitor group was significantly higher than the miR-186-5p inhibitor NC group, suggesting that miR-186-5p inhibitor enhanced the invasion of cancer cells. Simultaneously, the number of MDA-MB-231 cells crossing the basement membrane was dramatically reduced following transfection with miR-186-5p mimic, showing that the invasive capacity of MDA-MB-231, SUM159PT, JIMT-1, but not ZR-75-30 cells was significantly restricted.

### Overexpressed miR-186-5p regulated protein phosphorylation of the PI3K/AKT signaling pathway by degrading SBEM, inhibiting the migration and invasive ability of breast cancer cells

**Figure 6:**
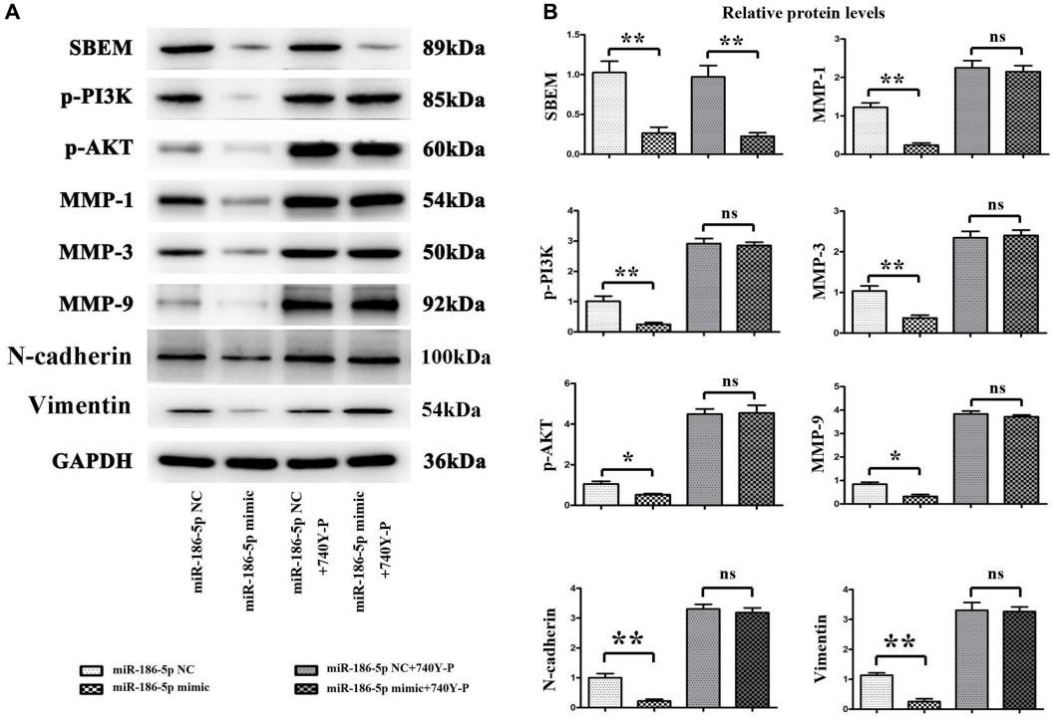
**Protein expression changes of SBEM, P-PI3K, p-Akt, T-P38, MMP1, MMP3 and MMP9, vimentin, N-cadherin in MDA-MB-231 cells in each group.** (**A**) Protein bands for SBEM, P-PI3K, p-Akt, T-P38, MMP1, MMP3 and MMP9, vimentin, N-cadherin; (**B**) Statistics on relative protein expression levels of SBEM, P-PI3K, p-Akt, T-P38, MMP1, MMP3 and MMP9, vimentin, N-cadherin. ^*^P < 0.05 and ^**^P < 0.01 indicated statistically significant differences.

The results of Western blotting (Figure 6A, 6B) showed that the expression of SBEM, p-PI3K and p-AKT proteins in MDA-MB-231 cells was significantly decreased after transfecting MDA-MB-231 cells with miR-186-5p mimic. Meanwhile, the relative expression level of MMP1, MMP3, and MMP9, related to the proliferation of cancer cells in the downstream pathway was significantly decreased. After adding 740Y-P, PI3K agonist, the expression of SBEM protein in cancer cells of miR-186-5p mimic + 740Y-P group was significantly decreased than the miR-186-5p mimic NC + 740Y-P group, while the protein levels of p-PI3K, p-AKT, MMP1, MMP3 and MMP9 were not significantly changed. These results suggested that miR-186-5p could degrade SBEM to regulate the PI3K/AKT signaling pathway, and exogenous overexpression of miR-186-5p could significantly inhibit the phosphorylation of proteins related to the PI3K/AKT signaling pathway and the migration and invasive ability of breast cancer cells.

### Overexpression of miR-186-5p inhibited the proliferation of breast cancer cells by regulating the PI3K/AKT signaling pathway

**Figure 7:**
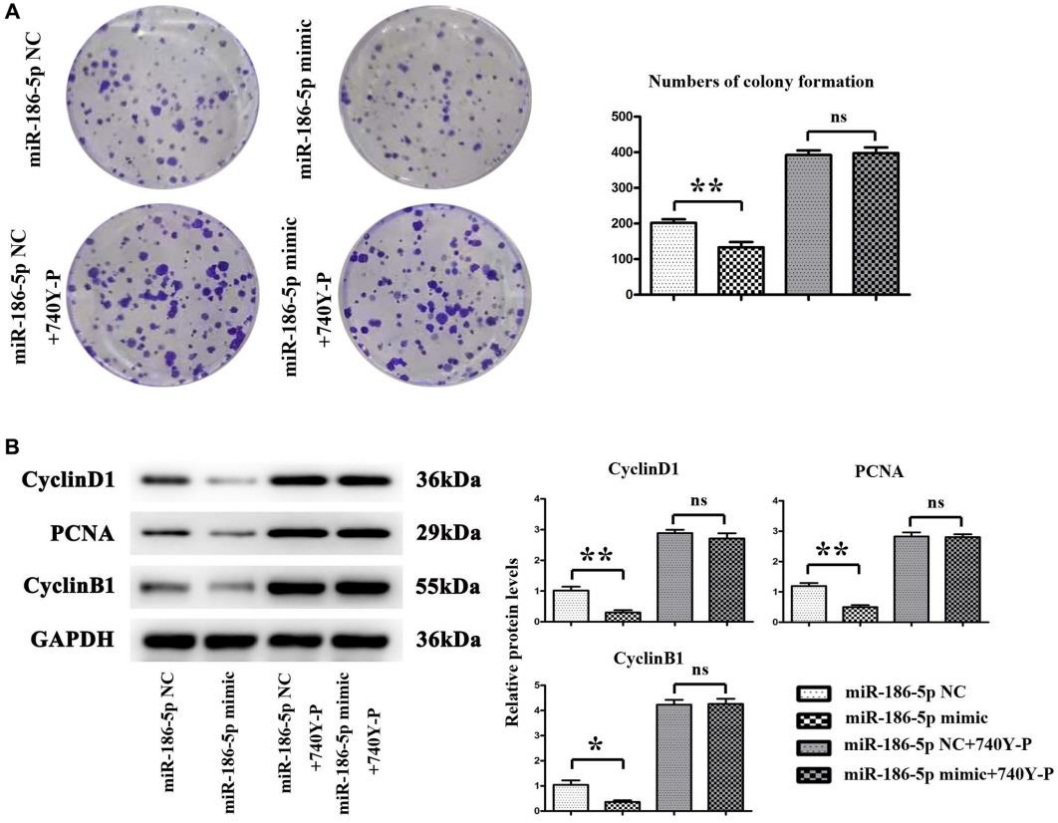
**Proliferation of MDA-MB-231 cells in each group.** (**A**) Changes in the number of colonies formed by mDA-MB-231 cells; (**B**) Protein expression levels of CyclinA2, CyclinD1 and PCNA in MDA-MB-231 cells. ^*^P < 0.05 and ^**^P < 0.01 indicated statistically significant differences.

The proliferation ability of MDA-MB-231 cells was evaluated by a monoclonal cell experiment. Figure 7A shows that the number of colonies formed by MDA-MB-231 cells in the miR-186-5p mimic group was significantly reduced than the miR-186-5p mimic NC group, suggesting that miR-186-5p could inhibit the proliferation ability of cancer cells. After adding 740Y-P, the number of colonies formed by MDA-MB-231 cells was not significantly changed. Meanwhile, the results were consistent with Western blotting, the expression of Cyclin D1, PCNA and Cyclin B1 proteins in MDA-MB-231 cells was significantly reduced in miR-186-5p mimic group than in miR-186-5p mimic NC group (Figure 7B). There was no significant difference in the protein expression between the two groups after adding 740Y-P, PI3K agonist, suggesting that exogenous overexpression of miR-186-5p could inhibit the proliferation of breast cancer cells by regulating the expression of cell proliferation-related proteins via PI3K-related pathway.

### Overexpression of miR-186-5p can inhibit the progression of breast cancer

**Figure 8:**
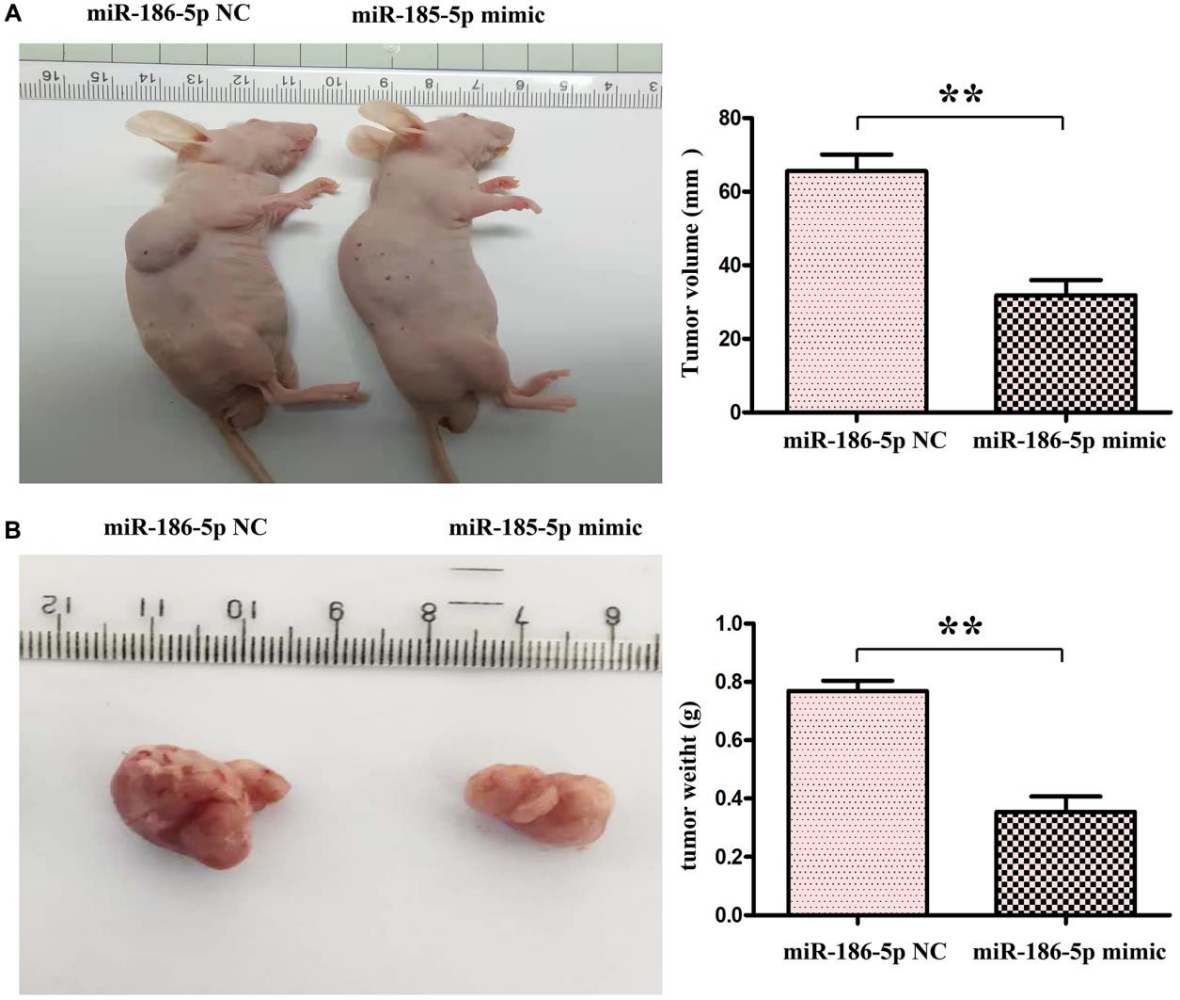
**miR-186-5p suppressed the progression of tumor in nude mice.** (**A**) Changes of tumor volumes in nude mice in miR-186-5p NC group and mimic group; (**B**) Changed of tumor weight in miR-186-5p NC group and mimic group; ^*^P < 0.05 and ^**^P < 0.01 indicated statistically significant differences vs. NC group.

**Figure 9:**
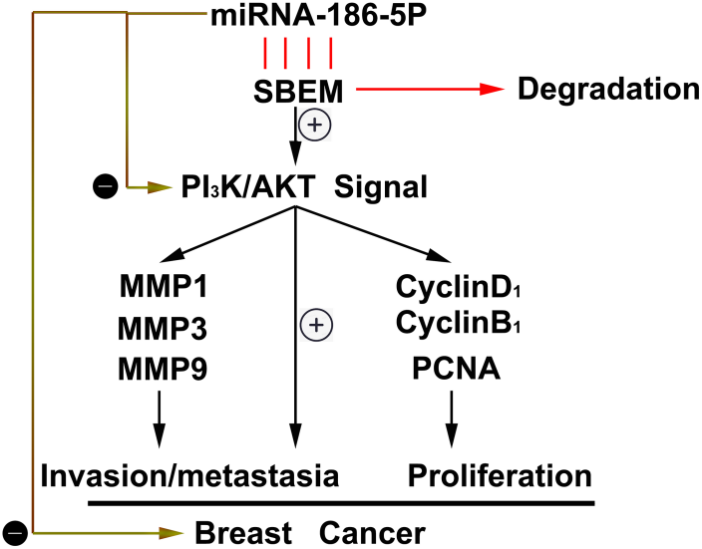
Thumbnail for the mechanisms of miR-186-5p on the progression of breast cancer.

Through the tumor bearing experiment in nude mice, we found that compared with the NC group, the tumor volume and tumor weight in the nude mice in the miR-186-5p mimic group were significantly reduced (Figure 8A, 8B). The above experiments show that miR186-5p binds to and degrades SBEM, thereby regulating the migration, invasion and proliferation of breast cancer cells by regulating the PI3K/Akt signaling pathway (Figure 9).

## DISCUSSION

Breast cancer is the most common malignant tumor globally, and its mortality is second only to lung cancer. In recent years, the incidence of breast cancer in China has been rapidly increasing. Dysregulation of miRNAs is a crucial step in the development, metastasis, angiogenesis, tolerance to chemotherapy, tolerance to radiation therapy, and stem cell maintenance of breast cancer [19, 20]. Numerous studies have identified differentially expressed miRNAs between normal and breast cancer tissue and established that these miRNAs are involved in the proliferation and metastasis of breast cancer cells via gene regulation. For example, miR-454-3p, miR-615-3p, etc., have been reported to be involved in breast cancer metastasis in different stages [21, 22]. However, the tissue-specific and condition-specific effect of miRNAs has seriously hindered the efficient translation of miRNAs into clinical cancer treatment [23]. Therefore, it is of great importance to investigate the role of miRNA in breast cancer metastasis and its molecular mechanism in the clinical intervention of breast cancer metastasis. In this study, we found that miR-186-5p was low expressed in breast cancer, and exogenous overexpression of miR186-5p significantly inhibited migration and invasion of breast cancer cells, suggesting that miR186-5p may inhibit the metastasis of breast cancer by targeting the expression of some genes.

SBEM (also known as MUCL1), which has been identified as a putative breast-specific gene, has been proposed to be a marker for predicting blood micrometastasis and response to neoadjuvant chemotherapy in breast cancer [24], is mainly expressed in breast and salivary glands, and is highly expressed in breast cancer tissues and metastatic lymph nodes [17]. Our results showed that SBEM had an abnormal high expression in breast cancer, accompanied by a low survival rate, suggesting that SBEM may be closely related to metastasis and invasion of cancer cells. In this study, differential genes were used to analyze the KEGG pathway and plot the KEGG pathway map, and it was concluded that the signaling pathways such as the PI3K-Akt signaling pathway and cell migration pathway were enriched. The expression of SBEM, p-PI3K, p-AKT, MMP1, MMP3, MMP9, CyclinD1, PCNA and CyclinB1 proteins in breast cancer cells transfected with miR-186-5p mimic was significantly decreased, and the proliferation of breast cancer cells was inhibited, indicating that miR-186-5p can degrade SBEM and inhibit phosphorylation of PI3K and AKT as well as the proliferation of cancer cells. MMPs play an important role in the occurrence and metastasis of breast cancer. They could destroy the local anatomical structure and stimulate the growth of tumor; and MMPs also destroys the basement membrane barrier, accelerates tumor metastasis and stimulates the formation of tumor neovascularization [25, 26]. The expression of N-cadherin contributes to angiogenesis and epithelial mesenchymal cell migration, and can also activate MAPK-ERK signal transduction pathway and induce MMP-9 gene expression, Thereby, it is conducive to the formation of tumor blood vessels and facilitate the tumor' grow and metastasize [27–30]. Vimentin is a type III intermediate filament protein expressed in stroma and an important marker of epithelial mesenchymal transition (EMT), an important factor leading to malignant tumor metastasis, and vimentin is an important protein to maintain the cytoskeleton structure. It is mainly expressed in mesenchymal cells and some ectodermal cells, which is closely related to cell movement [31, 32]. PCNA and cyclin family proteins are immune marker to detect the proliferative activity of tumor cells. Previous studies have be proved that the synthesis and expression of PCNA are related to the state of cell proliferation [33–35]. These proteins associated to tumor invasion and metastasis were under the modulation of PI3K/AKT signals [36–41]. The expression of SBEM protein in cancer cells of miR-186-5p mimic + 740Y-P group was significantly decreased than the miR-186-5p mimic NC + 740Y-P group, while the protein levels of p-PI3K, p-AKT, MMP1, MMP3 and MMP9 were not significantly changed, confirming that SBEM affects the expression of functional proteins related to cell migration, invasion and proliferation by regulating the phosphorylation of PI3K and AKT, thereby influencing the migration, invasion and proliferation of cancer cells.

To sum up, a new miRNA, namely miR186-5p is found to be low expressed in breast cancer tissues. As for the molecular mechanism, miR186-5p binds to and degrades SBEM, thereby acting on the migration, invasion, and proliferation of breast cancer cells by regulating the PI3K/Akt signaling pathway. However, whether the expression of miR186-5p in serum or plasma of breast cancer patients can be used to predict metastatic potential and prognosis of breast cancer needs to be explored.
